# Post-traumatic stress disorder and symptoms in paediatric cancer survivors and their family nucleus: systematic review, meta-analysis and meta-regression

**DOI:** 10.1192/bjo.2024.805

**Published:** 2024-11-11

**Authors:** Chen Ee Low, Sheryl Yen Pin Tan, Andre Loh, Jingrong Yu, Joel Zuo Er Ong, Caitlin Yuen Ling Loh, Chun En Yau, Ainsley Ryan Yan Bin Lee, Cyrus Su Hui Ho

**Affiliations:** Yong Loo Lin School of Medicine, National University of Singapore, Singapore; Department of Psychological Medicine, Yong Loo Lin School of Medicine, National University of Singapore, Singapore; and Department of Psychological Medicine, National University Hospital, Singapore

**Keywords:** Trauma and stressor-related disorders, systematic review, child and adolescent psychiatry, meta-analysis, carers

## Abstract

**Background:**

Various studies have highlighted the increased incidence and symptoms of depression and anxiety in paediatric cancer survivors (PCS). Yet no meta-analysis has focused on post-traumatic stress disorder (PTSD) or post-traumatic stress symptoms (PTSS) in PCS and their family nucleus.

**Aims:**

To evaluate the overall risk of PTSD and severity of PTSS in PCS and their family nucleus. Secondary objectives include identifying potential risk factors of PTSD and high PTSS.

**Method:**

We systematically searched PubMed, Embase and PsycINFO for studies comparing the risk of PTSD and PTSS severity among PCS, their family nucleus and non-cancer controls. PRISMA reporting guidelines were followed. Random effects meta-analyses and meta-regressions were conducted.

**Results:**

From 1089 records, we included 21 studies. PCS have an increased risk of PTSD (risk ratio 2.36, 95% CI 1.37–4.06) and decreased PTSS severity (standardised mean difference −0.29, 95% CI −0.50 to −0.08). Subgroup analyses of other categorical study-level characteristics revealed that female PCS who were older at diagnosis and data collection had a significantly higher risk of PTSD. Meta-regression were insignificant. Family nucleus did not show a significantly increased risk of PTSD (risk ratio 1.13, 95% CI 0.59–5.00) and PTSS severity (standardised mean difference 0.53, 95% CI −0.00 to 1.06). Systematically reviewing studies on the family nucleus found that the majority reported a significantly increased risk of psychological trauma compared with the comparator. Lower education, income and social status were also risk factors.

**Conclusions:**

Timely identification and interventions are imperative for policy makers and healthcare providers to prevent trauma from worsening in this population group.

In the 21st century, cancer remains one of the top causes of mortality.^1^ Every year, globally, there are an estimated 400 000 new cases of cancer in children and adolescents of up to 19 years old.^2^ Remarkable improvements in childhood cancer treatment and education have significantly increased both survival and attitudes toward adverse situations for the vast majority of childhood cancer survivors and their parents.^3^ However, paediatric cancer survivors may experience greater isolation from social activities than their peers, causing adverse psychosocial outcomes.^4^ Literature indicates that one of the most important psychological consequences for paediatric cancer survivors is post-traumatic stress disorder (PTSD).^5^ The risk of PTSD and mental health conditions has been shown to be much higher in paediatric cancer survivors, and results in significant morbidity.^6–8^

## Post-traumatic stress disorder and symptoms

Post-traumatic stress symptoms (PTSS) that constitute the diagnosis of PTSD have been a useful framework for understanding the incidence of PTSD in paediatric cancer survivors.^9^ Subclinical levels of PTSS are extremely common (up to 73.3%), and they cause serious consequences among paediatric cancer survivors and their families.^10^ For example, PTSD and PTSS can impair medical outcomes, greatly limit the quality of life and reduce educational and occupational achievement.^11^ It is widely known that childhood cancer-related PTSS is associated with neurocognitive deficits and psychiatric comorbidities.^12,13^ The treatment experience for paediatric cancer is a complex process that is time-consuming and exhausting.^14^ Different treatments, including chemotherapy, surgery and radiation, can cause long-term psychological side-effects.^15^ This could result from many reasons such as comorbidities, pain from disease, fear of death and the burden it places on their family. Others have proposed that biologically, exposure to cancer may increase one's vulnerability in expressing PTSS.^11^

High levels of PTSD and PTSS among children diagnosed with cancer have been shown to translate to a greater risk of psychiatric conditions in adulthood if left untreated.^16^ Without treatment, these conditions can turn chronic and affect normal psychosocial functioning in adulthood.^16^ A survey-based study found that one in five paediatric cancer survivors develop severe distress into adulthood that meet criteria for PTSD diagnosis.^17^ Therefore, there is a critical need to intervene during vulnerable periods after paediatric cancer survivors’ traumatic cancer experience.

## Family nucleus

Interest in the prevalence of PTSD and PTSS in the family nucleus of paediatric cancer survivors has risen in recent years. Because of the severe and distressing effects of cancer, young children rely heavily on their families during and after illness.^18^ These psychological consequences on the family members of paediatric cancer survivors could hamper their capacity to participate in important decision-making about their loved one's treatment, or even to provide emotional support.^19^ This could also deepen the effect of trauma on the paediatric cancer survivors. Thus, coupled with the various psychological, social and somatic difficulties that the family nucleus experiences,^20,21^ psychological trauma in the family nucleus is another complex issue to target.

## Objective

Various studies over the years have highlighted depression and anxiety incidence or symptoms in paediatric cancer survivors, with findings ranging from inconclusive, significantly reduced or increased risk compared with age-matched comparators. Previously, we explored the trends of PTSS in paediatric cancer survivors over time.^7^ We identified only two longitudinal studies evaluating PTSS in paediatric cancer survivors, and were not able to perform meta-analysis because of limited data. Both studies found that levels of PTSS remained consistently high up to 12 months after diagnosis. Detailed data on the incidence, severity and risk factors of PTSD and PTSS are already limited in paediatric cancer survivors, let alone their family nucleus. To the best of our knowledge, no studies have comprehensively focused on the risk factors, overall risk and severity of psychological trauma in both paediatric cancer survivors and their family nucleus. Hence, we aim to evaluate the overall risk of PTSD and the severity of psychological trauma in paediatric cancer survivors, as well as their family nucleus. Secondary objectives include identifying potential risk factors of PTSD and PTSS.

## Method

### Protocol and guidance

The systematic review is reported according to the Preferred Reporting Items for Systematic Reviews and Meta-Analyses (PRISMA) guidelines. Our protocol was registered prospectively on PROSPERO (reference: CRD42023413557).

### Definitions

In our review, paediatric patients are defined as those no older than 18 years old, following the definition of the United Nations.^22^ Paediatric cancer survivors are those who had a prior diagnosis of any solid or haematological cancer when they were no older than 18 years old and were currently in remission at the time of the study.^23^ PTSD is defined as a chronic impairment disorder that occurs after an exposure to traumatic events, which results in disturbance to functioning.^24^ PTSS constitutes the diagnosis of PTSD and includes a wide range of mental and physical symptoms, such as problems with concentration, sleep, increased reactivity, irritability, avoidance of traumatic triggers, hypervigilance, tachycardia and dizziness.^25^

### Data sources and search strategy

A literature search was performed in PubMed, EMBASE and PsycINFO. The search strategy combined search terms for paediatrics, cancer, and PTSD or PTSS. The database-controlled vocabulary was used for searching subject headings, and a large spectrum of synonyms with appropriate truncations was used for searching the title, abstract and author keywords. Given the development of trends in cancer epidemiology and cancer care, the search was limited to publications from 2000 to 26 August 2023. The full search strategies are available in Supplementary Table 1 available at https://doi.org/10.1192/bjo.2024.805.

### Study selection: inclusion and exclusion criteria

Two reviewers independently screened titles and abstracts of all studies for eligibility according to the inclusion and exclusion criteria. The full text of studies assessed as ‘relevant’ or ‘unclear’ was then independently evaluated by the same two reviewers. The interrater agreement was computed, and discrepancies were resolved with adjudication by a third independent reviewer.

**Fig. 1:**
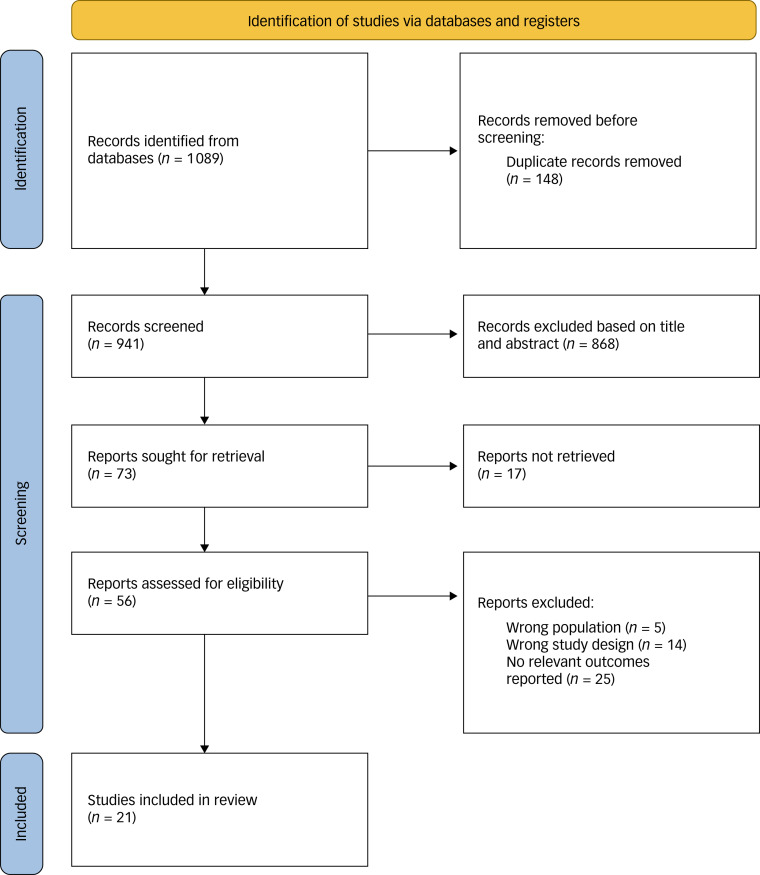
Preferred Reporting Items for Systematic Reviews and Meta-Analyses flowchart.

We included English-language, peer-reviewed studies published since 2000 that assessed the risk of PTSD or severity of PTSS following cancer diagnosis, and included paediatric cancer survivors or their family nucleus. We included studies that aimed to assess at least one of the following as a key finding: how a paediatric cancer diagnosis affected the risk of PTSD or severity of PTSS in both paediatric cancer survivors and their family nucleus. Non-empirical studies, non-controlled studies, grey literature, studies that did not stratify outcomes by age and studies only involving pharmacological or surgical intervention were excluded. The selection process is illustrated in Fig. 1.

### Data analysis

The *meta* and *metafor* packages on R (version 4.1.0 for MacOS, Posit PBC, USA; https://posit.co/download/rstudio-desktop/) were used to conduct all analyses. Unless specified, we considered a two-sided *P*-value of <0.05 as statistically significant. For continuous outcomes, in studies without s.d. values, confidence intervals were converted to s.d. To investigate the severity of PTSS, we pooled standardised mean differences (SMD). For dichotomous outcomes, we performed separate meta-analyses for the relative risk of PTSD (measured with risk ratios compared with non-cancer controls). Sensitivity analysis was conducted with the random-effects, leave-one-out analysis, identification and exclusion of potential outliers. Between-study heterogeneity was represented by *I*^2^ and τ^2^ statistics. An *I*^2^ of <30% indicated low heterogeneity between studies, 30–60% showed moderate heterogeneity and >60% indicated substantial heterogeneity.^26^

Subgroup analyses and meta-regression was performed to determine if key categorical and hierarchical variables influenced the results. We assessed for publication bias via visual inspection for funnel plot asymmetry and by using Egger's test. If publication bias was suspected, we conducted a sensitivity analysis with the trim-and-fill method (R0 estimator, fixed-random effects models) to re-estimate the pooled effect size after imputing potentially missing studies.^27,28^ This assumes a normal distribution of effect sizes around the centre of the funnel plot.^29^

### Risk-of-bias assessment

To assess methodological quality and the risk of bias of studies, we used the Joanna Briggs Institute (JBI) Critical Appraisal Checklist,^30^ which includes appraisal of the criteria for inclusion, measurement of condition, reporting of baseline characteristics, reporting of outcomes and appropriateness of the statistical analysis (if any).^31^ This appraisal was performed by two reviewers independently, with discrepancies resolved by the independent verdict of a senior reviewer.

## Results

### Overall population characteristics

From 1089 records, we included a total of 21 studies,^6,10,12,32–50^ resulting in 10 812 paediatric cancer survivors and their family nucleus, and 4765 non-cancer comparators (Fig. 1). Among the 21 studies, we evaluated and compared the country of study, the cancer type, the control group used for comparison and the scale used to evaluate symptoms of PTSD. There were 17 studies^6,10,12,32–45^ focusing on paediatric cancer survivors and eight studies investigating^32,36,38,42,47–50^ the family nucleus of paediatric cancer survivors. Four out of 21 studies^32,36,38,42^ looked at both paediatric cancer survivors and their family nucleus. The main characteristics of the included studies are summarised in Table 1.

**Table 1:** Characteristics of included studies of paediatric cancer survivors and their family nucleus

Study	Publication year	Country of study	Cancer type	Number at risk	Number of controls	Control characteristics	Proportion of males	Mean age at cancer diagnosis, years^a^	Mean age at follow-up/last outcome assessment, years^a^	Instrument, scales and diagnostic criteria for assessing PTSD and PTSS
Paediatric cancer survivors										
Barakat et al^12^	2000	USA	Various	56	65	Parents	Not reported	5.55 (3.74)	12.78 (3.73)	PTSD: PTSD-RI
Brown et al^32^	2003	USA	Various	52	42	Matched	0.44	9.42 (4.88)	17 (3.44)	PTSD: PTSD-RI
Gerhardt et al^33^	2007	USA	Various	56	60	Normative population data	0.66	7.29 (2.17)	18.63 (7.5)	PTSD: K-SADS
Phipps et al^34^	2014	USA	Various	255	101	Matched	0.52	12.7 (2.9)	12–18	PTSD: CAPS-CA
Schwartz and Drotar^35^	2006	USA	Various	57	83	Matched	0.47	13.60 (3.28)	21.7 (2.65)	PTSD: PCL-C
Yang et al^36^	2022	China	Various	91	114	Normative population data	0.63	10.92 (2.95)	6–18	PTSD: PTSD-RI, PTSS: PCL-C
Phipps et al^46^	2006	USA	Various	162	162	Matched	0.55	Not reported	12.9 (3.0)	PTSD: PTSD-RI, PCL-C
Kazak et al^10^	2004	USA	Various	220	252	Parents	0.48	7.9 (4.3)	14.7 (2.4)	PTSD: PTSD-RI
Seitz et al^37^	2010	Germany	Various	820	1027	Matched	0.49	15.78 (0.89)	30.4 (6.0)	PTSD: DSM-IV
Stuber et al^6^	2010	USA	Various	6542	368	Siblings	0.48	8.21 (5.87)	31.85 (7.55)	PTSD: SF-36
Tillery et al^38^	2019	USA	Various	50	47	Matched	0.55	2.15 (1.43)	4.57 (1.07)	PTSD: CBCL-PTSD
Bemis et al^39^	2015	USA	Various	151	318	Parents	0.53	10.6 (3.9)	13.5 (2.4)	PTSS: IES-R
Bruce^40^	2010	UK	Brain tumour	52	52	Parents	0.12	4–12	8–16	PTSS: IES-R
Clawson et al^42^	2013	USA	Various	119	108	Siblings	0.52	12.32 (3.43)	>18	PTSS: UCLA PTSD for DSM-IV
D'Urso et al^43^	2018	UK	Haematological	34	26	Siblings	0.38	Not reported	12.38 (2.85)	PTSS: IES-R
Ozono et al^44^	2007	Japan	Various	88	159	Parents	0.45	5.4 (3.8)	16.2 (2.2)	PTSS: IES-R
Phipps et al^45^	2009	USA	Various	199	108	Matched	0.52	Not reported	12.32 (3.4)	PTSS: DSM-IV
Family nucleus of paediatric cancer survivors										
Pöder^47^	2008	Sweden	Various	259	259	Spouse	0.53	7.8 (4.9)	Not reported	PTSD: PCL-C
Brown et al^32^	2003	USA	Various	52	42	Matched	0.00	9.42 (4.88)	17 (3.44)	PTSD: PTSD-RI
Baenziger et al^48^	2020	Switzerland	Various	663	391	Normative population data	0.55	6.8 (4.5)	23.9 (6.8)	PTSD: IES-R, PTSS: IES-R
McCarthy et al^49^	2021	Australia	Haematological	77	51	Matched	0.10	5 (3.17)	8.08 (3.15)	PTSD: PCL-S, PTSS: DASS-21
Tillery et al^38^	2019	USA	Various	50	47	Matched	0.14	2.15 (1.43)	4.57 (1.07)	PTSS: IES-R
Yang et al^36^	2022	China	Various	91	114	Normative population data	0.33	10.92 (2.95)	6–18	PTSS: PCL-C
Van Gorp et al^50^	2023	The Netherlands	Various	448	661	Normative population data	0.44	4.1 (3.0)	25.4 (3.5)	PTSS: SRS-PTSD
Clawson et al^42^	2013	USA	Various	168	108	Matched	0.08	12.32 (3.43)	>18	PTSS: UCLA PTSD for DSM-IV

PTSD, post-traumatic stress disorder; PTSS, post-traumatic stress symptoms; PTSD-RI, PTSD Reaction Index; K-SADS, Kiddie Schedule for Affective Disorders and Schizophrenia; CAPS-CA, Clinician-Administered PTSD Scale for Children and Adolescents; PCL-C, PTSD Checklist; SF-36, Short Form Survey 36 item; CBCL-PTSD, Child Behavior Checklist PTSD; IES-R, Impact of Event Scale Revised; UCLA, University of California, Los Angeles; PCL-S, PTSD Checklist Specific; DASS-21, Depression, Anxiety and Stress Scale 21 item; SRS-PTSD, Self-Rating Scale for PTSD.

a. Mean (s.d.) reported unless otherwise specified.

### Characteristics of the paediatric childhood survivors

Among the 17 studies on paediatric cancer survivors, most were from the USA,^6,10,12,32–35,38,39,42,45,46^ two studies were from the UK,^41,43^ one study was from China,^36^ one study was from Germany^37^ and one study was from Japan.^44^ All of the studies looked at paediatric cancer survivors with various cancer types, except one study on brain tumour survivors^41^ and one study on haematological cancer survivors.^43^ Seven studies^32,34,35,37,38,45,46^ recruited healthy, age-matched comparators as a control group, whereas five recruited parents,^10,12,39,41,44^ three recruited siblings^6,42,43^ and two utilised normative population data.^33,36^ The most prevalent scale used to evaluate symptoms of PTSD was the PTSD Reaction Index and Impact of Event Scale Revised (IES-R) for PTSS.

### Characteristics of the family nucleus of paediatric childhood survivors

Three out of the eight studies were from the USA,^32,38,42^ one was from China,^36^ one was from Sweden,^47^ one was from Australia,^49^ one was from The Netherlands^50^ and one was from Switzerland.^48^ All of the studies looked at paediatric cancer survivors with various cancer types, except one study on haematological cancer survivors.^49^ Three studies^36,48,50^ utilised normative population data as a control group, whereas four studies^32,38,42,49^ recruited healthy, age-matched comparators and one study recruited the spouse of an affected parent.^47^ The most prevalent scale used to measure symptoms of PTSD was the PTSD Checklist and IES-R for PTSS.

### Prevalence and risk of PTSD in paediatric cancer survivors

**Fig. 2:**
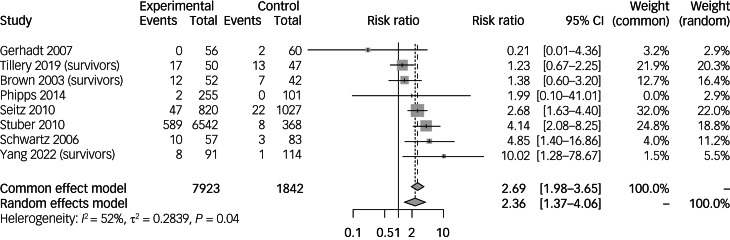
Incidence and risk ratios of post-traumatic stress disorder in paediatric cancer survivors compared with non-cancer controls. Survivors refers to studies with a population of paediatric cancer survivors.

Meta-analyses were performed to evaluate the risk of developing PTSD in paediatric cancer survivors (Fig. 2).^6,32–38^

Meta-analysis of 7923 paediatric cancer survivors compared with 1842 non-cancer controls showed that the risk of PTSD was significantly raised in paediatric cancer survivors (risk ratio 2.36, 95% CI 1.37–4.06). Four studies^6,35–37^ found the risk was significantly increased in paediatric cancer survivors. The other four studies found that the risk increased, although this did not reach statistical significance. Gerhardt et al^33^ found that the risk insignificantly decreased, but were limited by sample size, comparing 56 paediatric cancer survivors against 60 non-cancer controls.

Subgroup analyses of PTSD when stratified by various categorical variables are listed in Supplementary Table 2. Paediatric cancer survivors aged between 12 and 18 years at diagnosis (risk ratio 3.07, 95% CI 1.46–6.48) were more likely to have increased risk of PTSD than those aged between 8 and 12 years (risk ratio 2.93, 95% CI 1.43–5.98). Female paediatric cancer survivors had a significantly higher risk of PTSD (risk ratio 2.84, 95% CI 1.47–5.49). Additionally, there was a significant increase in the risk of PTSD found in paediatric cancer survivors aged above 18 years old at the time of data collection (risk ratio 2.97, 95% CI 1.24–7.12). Other categorical variables used were not found to significantly increase the risk of PTSD.

### Mean severity of PTSS in paediatric cancer survivors

**Fig. 3:**
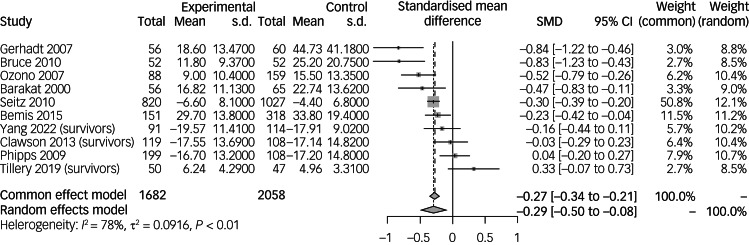
Mean severity of post-traumatic stress symptoms in paediatric cancer survivors compared with non-cancer controls. Survivors refers to studies with a population of paediatric cancer survivors. SMD, standardised mean difference.

Meta-analyses were performed to evaluate the mean severity of PTSS in paediatric cancer survivors^12,33,36–42,44,45^ (Fig. 3).

Meta-analysis of 1682 paediatric cancer survivors compared with 2058 non-cancer controls showed that the mean severity of PTSS was significantly decreased in paediatric cancer survivors (SMD = −0.29, 95% CI −0.50 to −0.08). Individually, all studies except two^38,45^ found that the severity of PTSS was significantly decreased.

Subgroup analyses of the severity of PTSS among other categorical variables are listed in Supplementary Table 3. Females paediatric cancer survivors were more likely to demonstrate a lower risk of PTSS (SMD = −0.50, 95% CI −0.79 to −0.21) compared with male paediatric cancer survivors (SMD = −0.14, 95% CI −0.38 to 0.09). Paediatric cancer survivors diagnosed between 2 and 8 years old were more likely to have less severe PTSS (SMD = −0.39, 95% CI −0.75 to −0.02) compared with those diagnosed between 8 and 12 years old (SMD = −0.38, 95% CI −0.78 to 0.02) or 12 and 18 years old (SMD = −0.10, 95% CI −0.49 to 0.28). Additionally, paediatric cancer survivors aged between 12 and 18 years old at the time of data collection had the highest likelihood of less severe PTSS (SMD = −0.35, 95% CI −0.65 to −0.04) compared with those aged between 2 and 12 years old (SMD = −0.25, 95% CI −0.81 to 0.32) or aged above 18 years (SMD = −0.17, 95% CI −0.68 to 0.33). Meta-regression of gender and age at diagnosis and data collection were not significant (Supplementary Table 4).

Studies that utilised parents as controls were more likely to demonstrate a decrease in severity of PTSS (SMD = −0.48, 95% CI −0.73 to −0.23) compared with age-matched controls (SMD = −0.07, 95% CI −0.31 to 0.17). The IES-R scale was more likely to pick up the decrease in severity of PTSS (SMD = −0.47, 95% CI −0.72 to −0.22) compared with the DSM-IV scale (SMD = −0.13, 95% CI −0.33 to 0.07). The test for subgroup differences revealed a significant difference between the type of controls (*P* = 0.002) and type of scale used (*P* < 0.01). Other categorical variables used were not found to significantly decrease the severity of PTSS.

### Prevalence and risk of PTSD in the family nucleus of paediatric cancer survivors

Four studies^32,47–49^ investigated associations between risk of PTSD in the family nucleus of paediatric cancer survivors (Table 2). Overall, two studies^32,49^ found a significant increase in risk of PTSD in the family nucleus compared with the comparison group. Among the family nucleus, three studies^42,48,49^ looked at both parents and one study^32^ focused on the mother.

**Table 2:** Evaluation of the mediating or confounding effect of the risk of post-traumatic stress disorder and mean severity of post-traumatic stress symptoms among the family nucleus of paediatric cancer survivors

Author	Publication year	Country of study	Study population	Key findings^a^
Risk of PTSD				
Brown et al^32^	2003	USA	52 mothers of PCS with various cancers, and 42 mothers of healthy children were recruited from a major university-affiliated medical centre in the USA. Mean age of PCS at diagnosis was 9.5 years, s.d. 4.88	Mothers of PCS reported significantly increased risk of PTSD than mothers of the comparison group (25% *v.* 7.1%; *P* < 0.001)
Baenziger et al^48^	2020	Switzerland	663 parents of PCS with various cancers (mean age 62.1 years, s.d. 6.8) and 391 parents of healthy children (mean age 61.8 years, s.d. 8) were studied as part of the nationwide Swiss Childhood Cancer Survivor Study. Mean age of PCS at diagnosis was 6.8 years, s.d. 4.5	Parents of PCS did not experience a significant increase in risk of PTSD compared with the comparison group (4.8% *v.* 6.7%; *P* = 0.210)
McCarthy et al^49^	2021	Australia	77 parents of PCS with haematological cancers (mean age 39.8 years, s.d. 5.2) and 52 parents of healthy children (mean age 41.7 years, s.d. 5.8) were recruited from the Royal Children's Hospital and Monash Children's Hospital in Australia from 2013 to 2017. Mean age of PCS at diagnosis was 5 years, s.d. 3.17	Parents of PCS experienced a significant increase in risk of PTSD compared with the comparison group (10% *v.* 0%; *P* < 0.05)
Clawson et al^42^	2013	USA	168 parents of PCS and 108 parents of the comparison cohort were recruited from out-patient clinics at a large paediatric oncology centre in the USA. Mean age of PCS was 12.32 years, s.d. 3.43	Chi-squared analysis revealed no significant differences in PTSD cases among the parents of PCS and parents of healthy control children (12.4% *v.* 9.8%; *P* = not significant)
Mean severity of PTSS				
Yang et al^36^	2022	China	91 parents of PCS with various cancers (mean age 38.24 years, s.d. 6.30) and 96 parents of healthy children (mean age 38.80 years, s.d. 4.67) were recruited from the paediatric departments of four general hospitals in southern China. Mean age of PCS at diagnosis was 10.92 years, s.d. 2.95	Parents of PCS (mean score 39.05, s.d. 13.45) reported significantly higher levels of PTSS than parents in the comparison group (mean score 24.48, s.d. 7.91; *P* < 0.001)
Pöder^47^	2008	Sweden	259 parents of PCS with various cancers (mean age at 37.9 years, s.d. 6.7) were recruited from Swedish paediatric oncology centres from 2002 to 2004. Mean age of PCS at diagnosis was 7.8 years, s.d. 4.9	Mothers reported consistently higher levels of PTSS compared with fathers (*P* < 0.001) across all three time points
Baenziger et al^48^	2020	Switzerland	663 parents of PCS with various cancers (mean age 62.1 years, s.d. 6.8) and 391 parents of healthy children (mean age 61.8 years, s.d. 8) were studied as part of the nationwide Swiss Childhood Cancer Survivor Study. Mean age of PCS at diagnosis was 6.8 years, s.d. 4.5	Parents of PCS did not experience a significant increase in severity of PTSS compared with the comparison group for intrusion (*P* = 0.322), avoidance (*P* = 0.078) and hyperarousal (*P* = 0.139)
McCarthy et al^49^	2021	Australia	77 parents of PCS with haematological cancers (mean age 39.8 years, s.d. 5.2) and 52 parents of healthy children (mean age 41.7 years, s.d. 5.8) were recruited from the Royal Children's Hospital and Monash Children's Hospital in Australia from 2013 to 2017. Mean age of PCS at diagnosis was 5 years, s.d. 3.17	Parents of PCS experienced a significant increase in severity of PTSS compared with the comparison group (17% *v.* 4%, *P* = 0.026)
Tillery et al^38^	2019	USA	50 parents of PCS with various cancers and 47 parents of healthy children were recruited from out-patient clinics at a St. Jude Children's Research Hospital. Mean age of PCS at diagnosis was 4.57 years, s.d. 1.07	Parents of PCS did not experience a significant increase in severity of PTSS compared with the comparison group (*P* = 0.24)
van Gorp et al^50^	2023	The Netherlands	448 parents of PCS with various cancers (mean age 56.5 years, s.d. 3.6) were studied as part of the Dutch Childhood Cancer Survivor Study LATER 2.	Mothers of PCS did not experience a significantly increased severity of PTSS compared with the fathers (mean score 2.3, s.d. 2.4 *v.* mean score 1.9, s.d. 2.2)
Clawson et al^42^	2013	USA	168 parents of PCS with various cancers and 79 parents of the comparison cohort were recruited from out-patient clinics at a large paediatric oncology centre in the USA. Mean age of PCS was 12.32 years, s.d. 3.43	Parents of PCS demonstrated a significantly increased severity of PTSS compared with the control group (*P* < 0.001)

PTSD, post-traumatic stress disorder; PTSS, post-traumatic stress symptoms; PCS, paediatric cancer survivors.

a. Mean (s.d.) reported unless otherwise specified.

Brown et al^32^ studied 52 mothers of paediatric cancer survivors, comparing the risk of PTSD to 42 age-matched controls. Results revealed that significantly more mothers of paediatric cancer survivors met the clinical criteria for PTSD diagnosis compared with mothers of the healthy comparison group (25% *v.* 7.1%; *P* < 0.001). Mothers of paediatric cancer survivors also reported higher PTSD symptom scores than their counterparts (*P* < 0.001). Baenziger et al^48^ found that there was no significant association in the prevalence of PTSD among 663 parents of paediatric cancer survivors and 391 comparison parents (4.8% *v.* 6.7%; *P* = 0.210). McCarthy et al^49^ observed 77 parents of paediatric cancer survivors compared with 52 parents with healthy children. Using the symptom cluster method of scoring with the Post-traumatic Stress Checklist Specific, there was a significantly higher prevalence of PTSD compared with the control group (10% *v.* 0%; *P* < 0.05). Clawson et al^42^ examined 168 parents of paediatric cancer survivors, comparing the risk of PTSD to 108 parents with healthy children. Although there were 34 total PTSD diagnoses made, they did not find any significant increase in risk of PTSD between the parents of paediatric cancer survivors and parents of healthy children. Further analysis found that parents of children who experienced a relapse had a higher prevalence of PTSD compared with parents of children with no relapse or parents of healthy children (27.6% *v.* 19.9% *v.* 9.8%; *P* = 0.047).

**Fig. 4:**
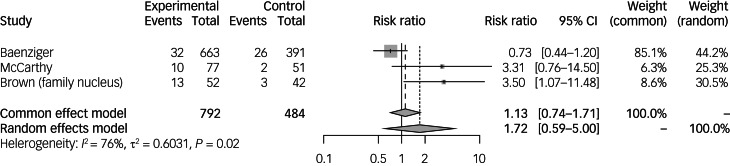
Incidence and risk ratios of post-traumatic stress disorder in the family nucleus of paediatric cancer survivors compared with non-cancer controls. Family nucleus refers to studies with a population of family nucleus of paediatric cancer survivors.

Meta-analysis was performed on 792 family nuclei of paediatric cancer survivors compared with 484 non-cancer controls^32,48,49^ (Fig. 4). The overall risk of PTSD in the family nucleus was not significantly increased (risk ratio 1.13, 95% CI 0.59–5.00). Individually, only Brown et al^32^ found that the risk of PTSD was significantly increased.

### Mean severity of PTSS in the family nucleus of paediatric cancer survivors

Seven studies^36,38,42,47–50^ looked at the severity of PTSS in the family nucleus of paediatric cancer survivors (Table 2). Overall, four out of seven studies^36,42,47,49^ found that the severity of PTSS in the family nucleus was significantly increased compared with the comparison group. Among the family nucleus, all of the studies focused on both parents, except two studies^47,50^ investigating only the mothers of paediatric cancer survivors.

Clawson et al^42^ studied 168 parents of paediatric cancer survivors diagnosed under the age of 18 years and compared the severity of PTSS to 79 age- and country-matched controls. Results revealed that parents of paediatric cancer survivors demonstrated a significantly increased severity of PTSS compared with parents of the healthy comparison group (*P* < 0.001). McCarthy et al^49^ found that 77 parents of paediatric cancer survivors experienced a significant increase in the severity of PTSS, compared with 52 comparison parents with healthy children (17% *v.* 4%; *P* = 0.026). Higher scores were also associated with being out of shape (49% *v.* 20%; *P* < 0.001) and poorer sleep (42% *v.* 26%; *P* = 0.004). Yang et al^36^ observed 91 parents of paediatric cancer survivors compared with 96 parents of healthy children. Parents of paediatric cancer survivors reported significantly higher PTSS severity than parents in the comparison group (*P* < 0.001). None of the demographic variables analysed, such as age, gender, time since cancer diagnosis and relapse status, were found to be significantly associated with PTSS severity. Tillery et al^38^ examined 50 parents of paediatric cancer survivors and did not find any significant increase in severity of PTSS (*P* = 0.24) when compared with 47 parents of healthy children. Analysis of age at diagnosis, time since diagnosis, treatment intensity, treatment status and relapse status did not reveal any significant predictors of PTSS severity.

**Fig. 5:**
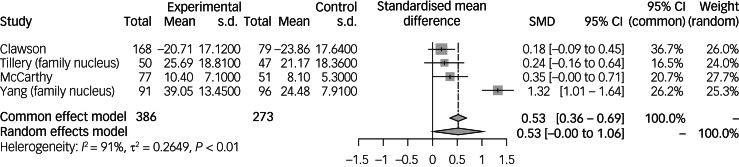
Mean severity of post-traumatic stress symptoms in the family nucleus of paediatric cancer survivors compared with non-cancer controls. Family nucleus refers to studies with a population of family nucleus of paediatric cancer survivors. SMD, standardised mean difference.

Meta-analysis of 386 family nuclei of paediatric cancer survivors compared with 273 non-cancer controls^36,38,42,49^ (Fig. 5) showed that risk was not significantly increased for mean severity of PTSS (SMD = 0.53, 95% CI −0.00 to 1.06). Individually, all of the studies found that the severity of PTSS was significantly increased in the family nucleus of paediatric cancer survivors.

### Education

Seven studies^6,33,35,37,39,48,49^ looked at the association between level of educational attainment and risk of PTSD and severity of PTSS (Supplementary Table 5). Out of the seven studies, five^6,33,35,37,39^ focused on paediatric cancer survivors and two^48,49^ looked at the family nucleus. Specifically, three^6,35,37^ out of the five studies on paediatric cancer survivors found significant association between lower educational attainment levels and increased risk of PTSD. The remaining two studies^33,39^ described no association between education level and risk of PTSD or severity of PTSS. Among the two studies on family nucleus, only one^48^ found a significant association between lower educational attainment and increased severity of PTSS.

### Social status and income level

Nine studies^6,32,35,37,41,43,45,48,49^ looked at the association between social status (marital or partnership status, employment status, social support and social integration) and risk of PTSD and severity of PTSS (Supplementary Table 6). Among the nine studies, six^6,35,37,41,43,45^ looked at paediatric cancer survivors, two^48,49^ focused on the family nucleus and one investigated both populations.^32^ Five^6,32,35,37,43^ studies on paediatric cancer survivors found a significant association between having a better social status and increased risk of PTSD and higher severity of PTSS. Those who were employed, had partners and positive social support had lower levels of PTSD or decreased severity of PTSS. Out of the three studies on the family nucleus, only one^49^ did not find any significant association. Brown et al^32^ found that having greater social support reduced risk of PTSD in mothers, and Baenziger et al^48^ observed that being in a partnership resulted in lower severity of PTSS.

Eight studies^6,32,33,36,38,39,45,46^ investigated associations between income level and risk of PTSD and severity of PTSS (Supplementary Table 7). Overall, five studies^6,33,39,45,46^ focused on paediatric cancer survivors, two^32,36^ looked at the family nucleus and one^38^ investigated both populations. Two studies^6,39^ on paediatric cancer survivors found a significant association between lower income levels and increased risk of PTSD and higher severity of PTSS. Among the three studies on the family nucleus, only one^36^ reported a significant association between lower income levels and increased severity of parental PTSS.

### Risk of bias, publication bias and sensitivity analyses

The quality of the methodologies of the 21 studies included in the meta-analysis, as scored with the JBI Checklist, is presented in Supplementary Table 8. Overall, no significant risk of bias in the studies was identified. Sensitivity analyses, funnel plots, trim-and-fill method and Egger's test showed some publication bias (Supplementary Figs 1–10).

## Discussion

Our results suggest that a paediatric cancer diagnosis is significantly associated with an increased risk of PTSD and lower severity of PTSS compared with non-cancer controls. Subgroup analyses revealed that paediatric cancer survivors who were female and older at the time of diagnosis and data collection had a significantly higher risk of PTSD. Additionally, paediatric cancer survivors who were female and younger at the time of diagnosis had the highest likelihood of decreased severity of PTSS. Family nucleus of paediatric cancer survivors did not demonstrate a significantly increased risk of PTSD and severity of PTSS. Systematically reviewing the studies on the family nucleus found that the majority reported a significantly increased risk of PTSD and severity of PTSS when compared with the comparator arm. Included studies investigated patients with a range of characteristics, including gender, control type, age at diagnosis and data collection, scales used, as well as social, cultural and economic backgrounds. To the best of our knowledge, our study is the first systematic review and meta-analysis to elucidate the burden of a paediatric cancer diagnosis on the risk of PTSD and severity of PTSS in both paediatric cancer survivors and their family nucleus.

Although a large majority of cancer patients are older adults,^51^ the incidence of cancer in the paediatric population is rapidly rising.^52,53^ These children are predisposed to psychosocial problems such as depression and anxiety, which may require complex treatments.^54,55^ The trauma that a cancer diagnosis and treatment can cause to a patient has been well studied. Yet, there is relatively scarce literature on the negative repercussions and long-term effects on paediatric cancer survivors and their family nucleus.

Our study demonstrated that paediatric cancer survivors have an increased risk of PTSD and lower severity of PTSS compared with controls. A younger age is consistently associated with higher rates of psychiatric syndromes and distress.^56^ Paediatric patients tend to present with more aggressive disease compared with older adults, likely because of limited treatment options and higher treatment burden.^57^ Furthermore, multiple studies^58–60^ have demonstrated that throughout all cancer phases, a younger age is associated with greater cancer pain, more negative outlook, poorer quality of life and higher concerns about sexuality, body image and fertility. Allen et al^61^ conducted a cohort study and found that one in eight long-term survivors of childhood cancer had PTSD, compared with an insignificant rate among the long-term adult cancer population. It may seem counterintuitive that paediatric cancer survivors have higher levels of PTSD, but lower severity of PTSS. However, in the natural course of adjustment to the cancer, it is expected that PTSS decrease over time.^62,63^ This has been shown in other psychological conditions, such as anxiety.^64^ Post-traumatic growth could also explain this phenomenon, where individuals experience a positive psychological change after struggling with a difficult life circumstances.^65^ Another possible explanation for the apparently decreased PTSS could be that most of the studies recruited family members as the non-cancer controls. Family members may be prone to more psychological stress after witnessing the struggles of their loved ones. Future studies should recruit matched controls who have experienced other forms of psychological trauma.

Various factors, such as age at diagnosis or data collection and gender, were found to significantly affect psychological trauma in paediatric cancer survivors. Alderfer et al explained that worsening levels of PTSD with age could be a result of the cumulative recollection of the situation, greater understanding of the realities of cancer, and societal pressures and norms that they interacted with.^66^ The mental pressure could potentially put them at a higher risk compared with younger cancer survivors. Another hypothesis on differing PTSS scores with age could be attributable to delayed neurodevelopment effects on the hippocampus and amygdala.^67^ During the early stages of life, centres of the brain responsible for emotional response and regulation are known to be adaptable.^68^ Even if the symptoms do not appear immediately after the event, experiencing trauma or a series of traumatic events over time could heighten an individual's vulnerability to psychopathology.^69^ Our study also found that females experienced greater risk of PTSD and severity of PTSS. Similar findings have been replicated across different international studies.^70–72^ Some of the possible explanations include having a more sensitive hypothalamus-pituitary-axis compared with men,^73^ where amygdala hyperactivity has been shown to be associated with PTSD,^74^ and females having a higher amount of empathy.^75^

Studies that recruited siblings or parents of the paediatric cancer survivors allowed valuable insights into the development of psychological trauma within the family. These studies revealed that levels of PTSD and PTSS among siblings and parents were higher than the general population, although statistically insignificant. Like the paediatric cancer survivors, family members commonly experience psychological trauma.^62^ Family members could be exposed to multiple potentially traumatic events during the treatment of paediatric cancer survivors. Those may include the diagnosis process, hospital admissions/stays, seeing their loved one in pain, adverse effects of treatment, fear of losing their loved one and deaths of other patients.^76^ However, family dynamics and other factors could affect how every family handles a traumatic event.^77^ A nationwide cross-sectional study by Baenziger et al^48^ found no increased risk of PTSS among parents of paediatric cancer survivors compared with parents within the general population. Future research should focus on understanding and supporting the entire family to help manage and resolve such symptoms in the long term.

Lower levels of educational attainment, income and social status were also found to be significant risk factors for PTSD and PTSS in paediatric cancer survivors and their family nucleus. These risk factors are well-studied and consistent across studies globally.^78–80^ With the increase in the burden of cancer^41^ and worsening challenges of inequality,^81^ our findings serve to highlight the importance of allocating resources to enhance effective detection and prevention strategies. This may guide future active and passive surveillance in these vulnerable subgroups. Across the age spectrum, a diagnosis of cancer is known to result in significant accompanying morbidity and poorer treatment outcomes.^82,83^ To directly address the needs of paediatric cancer survivors and their family nucleus, psycho-oncological interventions should be developed to capitalise on these risk factors.

### Limitations

Our review faced several limitations. First, the observed associations in our study were subjected to substantial heterogeneity across studies, which varied across countries and populations, spanning a range of sociocultural and economic backgrounds. We anticipated heterogeneity in methods of defining and assessing these variables, and hence we adopted the synthesis without meta-analysis approach. Second, the instruments and questionnaires that quantified PTSS burden had a degree of heterogeneity. Although these validated instruments evaluated similar domains, there remains heterogeneity that may not be accounted for. Finally, the assessment of risk factors could be less granular, as we did not obtain individual patient data for our meta-analysis. To overcome this, we systematically synthesised the individual analyses performed by each study, to identify vulnerability factors.

In conclusion, we elucidated a significantly increased risk of psychological trauma among paediatric cancer survivors. The family nucleus of paediatric cancer survivors was not at significantly increased risk of psychological trauma. A diagnosis of cancer is not only associated with physical burden, but also a lasting psychological impact on the patient, family and social units. If unaddressed, high levels of this psychological trauma may translate to a greater risk of psychiatric comorbidities during adulthood. Most importantly, timely identification and intervention is imperative for both policy makers and healthcare providers, to prevent worsening of PTSD and PTSS among paediatric cancer survivors and their family nucleus.

## Supporting information

Low et al. supplementary material Low et al. supplementary material

## Data Availability

Data availability is not applicable to this article as no new data were created or analysed in this study.
