# Supplementary material for: Post-traumatic stress disorder and symptoms in paediatric cancer survivors and their family nucleus: systematic review, meta-analysis and meta-regression
Source: BJPsych Open. 2024 Nov 11;10(6):e207. doi: 10.1192/bjo.2024.805 (PMC11698174; doi:10.1192/bjo.2024.805)

**Supplementary Materials**

**Supplementary Table 1:** Search strategy

**Supplementary Table 2:** Meta-analyses of PTSD in PCS stratified by categorical study-level characteristics using the random effect model

**Supplementary Table 3:** Meta-analyses of the severity of PTSS in PCS stratified by categorical study-level characteristics using the random effect model

**Supplementary Table 4:** Mixed effects meta-regression of standardised mean differences against potential effect moderators (continuous and categorical study-level characteristics) for the severity of PTSS in PCS

**Supplementary Table 5:** Evaluation of the mediating or confounding effect of educational attainment level of participants on risk of PTSD and severity of PTSS **Supplementary Table 6:** Evaluation of the mediating or confounding effect of social status of participants on risk of PTSD and severity of PTSS

**Supplementary Table 7:** Evaluation of the mediating or confounding effect of income level of participants on risk of PTSD and severity of PTSS

**Supplementary Table 8:** Quality assessment of included cohort studies using the Joanna Brigg’s Institute Critical Appraisal tool

**Supplementary Figure 1:** Funnel plot for visual inspection of publication bias in studies assessing risk of PTSD in PCS

**Supplementary Figure 2:** Trim-and-fill analysis for publication bias in studies assessing risk of PTSD in PCS

**Supplementary Figure 3:** Quantitative assessment publication bias in studies assessing risk of PTSD in PCS

**Supplementary Figure 4:** Leave-one-out analysis of studies assessing the risk of PTSD in PCS, compared to non-cancer controls, using the random effects model

**Supplementary Figure 5:** Outlier assessment of studies assessing the risk of PTSD in PCS, compared to non-cancer controls, using the random effects model

**Supplementary Figure 6:** Funnel plot for visual inspection of publication bias in studies assessing severity of PTSS in PCS

**Supplementary Figure 7:** Trim-and-fill analysis for publication bias in studies assessing severity of PTSS in PCS

**Supplementary Figure 8:** Quantitative assessment publication bias in studies assessing severity of PTSS in PCS **Supplementary Figure 9:** Leave-one-out analysis of studies assessing the severity of PTSS in PCS, compared to non-cancer controls, using the random effects model

**Supplementary Figure 10:** Outlier assessment of studies assessing the risk of PTSD of PCS in PCS, compared to non-cancer controls, using the random effects model

**Supplementary Table 1:** Search strategy

**EMBASE**

| (Pediatric* OR paediatric* OR juvenile* OR “young adult*” OR ‘childhood’):ti,ab   AND  "neoplasm"/exp or (**‘**neoplasm' OR 'cancer*' OR 'tumo*' OR 'malign*' OR ‘leukemia*’ OR ‘lymphoma*’):ti,ab  AND  ('posttraumatic stress disorder’ OR ‘posttraumatic stress’ OR ‘ptsd’ OR ‘post-traumatic’ OR ‘post traumatic’ OR ‘traumatic stress*’)/exp  AND  NOT [medline]/lim |
| --- |

**PubMed**

| ("Pediatric*"[Title/Abstract] OR "paediatric*"[Title/Abstract] OR "Young Adult"[Title/Abstract] OR "Child*"[Title/Abstract] OR juvenile*[Title/Abstract] OR infant*[Title/Abstract] OR adolesc*[Title/Abstract])  AND  (neoplas*[Title/Abstract] OR cancer*[Title/Abstract] OR tumo*[Title/Abstract] OR malign*[Title/Abstract] OR leukemia*[Title/Abstract] OR lymphoma*[Title/Abstract])  AND  ("Post-Traumatic*"[Title/Abstract] OR "Post Traumatic Stress*"[Title/Abstract] OR "PTSD"[Title/Abstract] OR "posttraumatic*"[Title/Abstract] OR "Post Traumatic*"[Title/Abstract]) |
| --- |

**APA PsycINFO**

| exp Pediatrics/ or exp Young Adulthood/ or exp Young Adult/ or exp Infant/ or paediatric*.mp. or juvenile*.mp. or childhood.mp. or child.mp.  AND  exp Cancers/ or exp Malignant Neoplasms/ or exp Neoplasms/ or exp Benign Neoplasms/ or exp Leukemias/ or exp Metastasis/ or exp Tumors/ or Lymphoma.mp. or malign*.mp. or tumo*.mp.  AND  ("Post-Traumatic*"[Title/Abstract] OR "Post Traumatic Stress*"[Title/Abstract] OR "PTSD"[Title/Abstract] OR "posttraumatic*"[Title/Abstract] OR "Post Traumatic*"[Title/Abstract])  AND  exp PTSD/ or Post Traumatic Stress Disorder.mp. or Post Traumatic Stress.mp. OR posttraumatic.mp. OR Post Traumatic.mp. |
| --- |

**Supplementary Table 2**: Meta-analyses of PTSD in PCS stratified by categorical study-level characteristics using the random effect model

| **Variable** | **Cohorts** | **N, cancer** | **N, control** | **RR** | **95% CI** | **I2** | **Test of interaction (p-value)** |
| --- | --- | --- | --- | --- | --- | --- | --- |
| Overall | 8 | 7923 | 1842 | 2.36 | 1.37; 4.06 | 52% | NA |
| Matched control | 5 | 1234 | 1300 | 2.04 | 0.95; 4.38 | 38% | 0.72 |
| General population | 2 | 147 | 174 | 2.53 | 0.35; 18.29 | 77% |  |
| Siblings | 1 | 6542 | 368 | 4.14 | 2.08; 8.25 | NA |  |
| Age at diagnosis between 2 to 8 | 2 | 106 | 107 | 1.05 | 0.42; 2.65 | 19% | 0.15 |
| Age at diagnosis between 8 to 12 | 3 | 6685 | 524 | 2.93 | **1.43; 5.98** | 63% |  |
| Age at diagnosis between 12 to 18 | 3 | 1132 | 1211 | 3.07 | **1.46; 6.48** | 0% |  |
| Age at data collection between 2 to 12 | 2 | 141 | 161 | 2.14 | 0.60; 7.61 | 73% | 0.71 |
| Age at data collection between 12 to 18 | 2 | 307 | 143 | 1.48 | 0.35; 6.21 | 0% |  |
| Age at data collection >18 | 4 | 7475 | 1538 | 2.97 | **1.24; 7.12** | 34% |  |
| Proportion of males <50% | 4 | 7471 | 1520 | 2.84 | **1.47; 5.49** | 37% | 0.33 |
| Proportion of males >50% | 4 | 452 | 322 | 1.58 | 0.60; 4.17 | 43% |  |

Abbreviations: RR, risk ratio; NA, not applicable; CI, confidence interval

**Supplementary Table 3**: Meta-analyses of the severity of PTSS in PCS stratified by categorical study-level characteristics using the random effect model

| **Variable** | **Cohorts** | **N, cancer** | **N, control** | **RR** | **95% CI** | **I2** | **Test of interaction (p-value)** |
| --- | --- | --- | --- | --- | --- | --- | --- |
| Overall | 10 | 1682 | 2058 | -0.29 | **-0.50; -0.08** | 78% | NA |
| Matched control | 4 | 1160 | 1296 | -0.07 | -0.31; 0.17 | 80% | **0.02** |
| Parents | 4 | 347 | 594 | -0.48 | **-0.73; -0.23** | 64% |  |
| Siblings | 1 | 119 | 108 | -0.03 | -0.29; 0.23 | NA |  |
| General population | 1 | 56 | 60 | -0.84 | -1.22; -0.46 | NA |  |
| Age at diagnosis between 2 to 8 | 4 | 250 | 331 | -0.39 | **-0.75; -0.02** | 84% | 0.51 |
| Age at diagnosis between 8 to 12 | 3 | 294 | 484 | -0.38 | -0.78; 0.02 | 75% |  |
| Age at diagnosis between 12 to 18 | 3 | 1138 | 1243 | -0.10 | -0.49; 0.28 | 78% |  |
| Age at data collection between 2 to 12 | 2 | 102 | 99 | -0.25 | -0.81; 0.32 | 94% | 0.83 |
| Age at data collection between 12 to 18 | 6 | 641 | 824 | -0.35 | **-0.65; -0.04** | 76% |  |
| Age at data collection >18 | 2 | 939 | 1135 | -0.17 | -0.68; 0.33 | 72% |  |
| Proportion of males <50% | 4 | 1016 | 1303 | -0.50 | **-0.79; -0.21** | 66% | 0.06 |
| Proportion of males >50% | 6 | 666 | 755 | -0.14 | -0.38; 0.09 | 77% |  |
| IES-R | 3 | 291 | 529 | -0.47 | **-0.72; -0.22** | 75% | **<0.01** |
| DSM-IV | 4 | 1229 | 1357 | -0.13 | -0.33; 0.07 | 68% |  |
| K-SADS | 1 | 56 | 60 | -0.84 | -1.22; -0.46 | NA |  |
| PTSD-RI | 1 | 56 | 65 | -0.47 | -0.83; -0.11 | NA |  |
| CBCL-PTSD | 1 | 50 | 47 | 0.33 | -0.07; 0.73 | NA |  |

Abbreviations: RR, risk ratio; NA, not applicable; CI, confidence interval

**Supplementary Table 4:** Mixed effects meta-regression of standardised mean differences against potential effect moderators (continuous and categorical study-level characteristics) for the severity of PTSS in PCS

|  | **Ratio** | **P** | **95% CI Lower** | **95% CI Upper** | **I^2^ (% residual heterogeneity)** |
| --- | --- | --- | --- | --- | --- |
| Age at diagnosis between 2 to 8 | -0.2812 | 0.2960 | -0.8086 | 0.2462 | 85.82% |
| Age at diagnosis between 8 to 12 | -0.2750 | 0.3323 | -0.8311 | 0.2810 |  |
| Age at data collection between 1 to 12 | -0.0774 | 0.8411 | -0.8347 | 0.6798 | 87.32% |
| Age at data collection between 12 to 18 | -0.1794 | 0.5522 | -0.7695 | 0.4113 |  |
| Proportion of males >50% | 0.3562 | 0.0618 | -0.0176 | 0.7299 | 78.65% |

Abbreviations: CI, confidence interval

**Supplementary Table 5:** Evaluation of the mediating or confounding effect of educational attainment level of participants on risk of PTSD and severity of PTSS

| **Author** | **Year** | **Country** | **Study population** | **Key findings†** |
| --- | --- | --- | --- | --- |
| **Paediatric Cancer Survivors** | | | | |
| Gerhardt | 2007 | USA | 56 PCS with various cancers, mean age at diagnosis 7.29 years, SD 2.17, were recruited as part of a longitudinal study of family adjustment to childhood cancer. | There was no significant association found between parental education levels and increased risk of PTSD among PCS (p= ns). |
| Schwartz | 2006 | USA | 57 PCS with various cancers, mean age at data collection 21.70 years, SD 2.65 were recruited by the division of oncology at a Midwest children’s hospital. Participants had to have a history of cancer between ages of 4 to 18 and are currently in remission. | Significant association found between lower education levels and higher risk of PTSD among PCS (p<0.01). |
| Seitz | 2010 | Germany | 820 PCS with various cancers, mean age at diagnosis 15.78 years, SD 0.89 were recruited from the German Childhood Cancer Registry from January 2008 to February 2009. | Significant association found between education levels and higher risk of PTSD among PCS (p<0.05). |
| Stuber | 2010 | USA | 6542 PCS with various cancers, mean age at diagnosis 8.21 years, SD 5.87 were recruited as part of the Childhood Cancer Survivor Study. | Significant association found between those with education levels of high school or less and higher risk of PTSD among PCS (95% CI = 1.16; 1.98). |
| Bemis | 2015 | USA | 151 PCS with various cancers, mean age at diagnosis 10.6 years, SD 3.9 were recruited from two paediatric hospitals in the US. | There was no significant association found between education levels and severity of PTSS among PCS as compared to control (p<0.01). |
| **Family Nucleus of Paediatric Cancer Survivors** | | | | |
| Baenziger | 2020 | Switzerland | 663 parents of PCS with various cancers, mean age 62.1 years, SD 6.8 and 391 parents of healthy children, mean age 61.8 years, SD 8, were studied as part of the nationwide Swiss Childhood Cancer Survivor Study. Mean age of PCS at diagnosis was 6.8 years, SD 4.5. | Significant association found between lower education levels and more characteristics (intrusion, avoidance, and hyperarousal) of increased severity of PTSS (p<0.002). |
| McCarthy | 2021 | Australia | 77 parents of PCS with haematological cancers, mean age 39.8 years, SD 5.2 and 52 parents of health children, mean age 41.7 years, SD 5.8, were recruited from the Royal Children’s Hospital and Monash Children’s Hospital in Australia from 2013 to 2017. Mean age of PCS at diagnosis was 5 years, SD 3.17. | No significant association found between education levels and severity of parental PTSS (95% CI -5.38; 3.42). |

Abbreviations: Post-traumatic Stress Disorder, PTSD; Post-traumatic Stress Symptom, PTSS; Paediatric Cancer Survivors, PCS; Standard Deviation, SD; Confidence Interval, CI; Not Significant, NS.
†Outcomes of interest include logistic or linear regression analysis for any association between education level and risk of PTSD and severity of PTSS.

**Supplementary Table 6:** Evaluation of the mediating or confounding effect of social status of participants on risk of PTSD and severity of PTSS

| **Author** | **Year** | **Country** | **Study population** | **Key findings†** |
| --- | --- | --- | --- | --- |
| **Paediatric Cancer Survivors** | | | | |
| Brown | 2003 | USA | 52 PCS with various cancers, mean age at diagnosis 9.42 years, SD 4.88, were recruited from a major-university affiliated medical center. | Significant association found between those with lesser social support and higher risk of PTSD among PCS (p<0.05). |
| Schwartz | 2006 | USA | 57 PCS with various cancers, mean age at data collection 21.70 years, SD 2.65 were recruited by the division of oncology at a Midwest children’s hospital. Participants had to have a history of cancer between ages of 4 to 18 and are currently in remission. | Significant association found between PCS with positive social support and lower risk of PTSD (p<0.01). |
| Seitz | 2010 | Germany | 820 PCS with various cancers, mean age at diagnosis 15.78 years, SD 0.89 were recruited from the German Childhood Cancer Registry from January 2008 to February 2009. | Significant association found between social status and PTSD among PCS (p<0.05). |
| Stuber | 2010 | USA | 6542 PCS with various cancers, mean age at diagnosis 8.21 years, SD 5.87 were recruited as part of the Childhood Cancer Survivor Study. | Significant association found between being single (95% CI 1.58; 2.50), unemployed (95% CI 1.62; 2.51) and higher levels of PTSD. |
| Bruce | 2011 | UK | 52 PCS with brain tumours, age at diagnosis between 8 to 16 years were recruited at a single-site children’s hospital in the UK. | No significant association between social environment and rates of PTSS in PCS and parents. (p<0.05). |
| D'Urso | 2018 | UK | 34 PCS with haematological cancers, mean age at diagnosis 12.38 years, SD 2.85 were recruited by a hospital’s paediatric oncology department in the UK. | Significant association found between perceived social support and decreased severity of PTSS in the control group (p<0.05). |
| Phipps | 2009 | USA | 199 PCS with various cancers, mean age at data collection 12.38 years, SD 3 were recruited from outpatient clinics at a major paediatric oncology center in the USA. | No significant association found between social status and severity of PTSS score (P>0.20). |
| **Family Nucleus of Paediatric Cancer Survivors** | | | | |
| Brown | 2003 | USA | 52 mothers of PCS with various cancers, and 42 mothers of healthy children were recruited from a major-university affiliated medical centre in USA. Mean age of PCS at diagnosis was 9.5 years, SD 4.88. | Significant association found between greater social support and lesser risk of PTSD symptoms in mothers (P<0.05). |
| Baenziger | 2020 | Switzerland | 663 parents of PCS with various cancers, mean age 62.1 years, SD 6.8 and 391 parents of healthy children, mean age 61.8 years, SD 8, were studied as part of the nationwide Swiss Childhood Cancer Survivor Study. Mean age of PCS at diagnosis was 6.8 years, SD 4.5. | Significant association found between being in a partnership and having a lower PTSS score (p=0.01). |
| McCarthy | 2021 | Australia | 77 parents of PCS with haematological cancers, mean age 39.8 years, SD 5.2 and 52 parents of health children, mean age 41.7 years, SD 5.8, were recruited from the Royal Children’s Hospital and Monash Children’s Hospital in Australia from 2013 to 2017. Mean age of PCS at diagnosis was 5 years, SD 3.17. | No significant association found between social risk factors and severity of PTSS among control group (95% CI, 8.61; 15.51). |

Abbreviations: Post-traumatic Stress Disorder, PTSD; Post-traumatic Stress Symptom, PTSS; Paediatric Cancer Survivors, PCS; Standard Deviation, SD; Confidence Interval, CI.
†Outcomes of interest include logistic or linear regression analysis for any association between social status and risk of PTSD and severity of PTSS

**Supplementary Table 7:** Evaluation of the mediating or confounding effect of income level of participants on risk of PTSD and severity of PTSS

| **Author** | **Year** | **Country** | **Study population** | **Key findings†** |
| --- | --- | --- | --- | --- |
| **Paediatric Cancer Survivors** | | | | |
| Gerhardt | 2007 | USA | 56 PCS with various cancers, mean age at diagnosis 7.29 years, SD 2.17, were recruited as part of a longitudinal study of family adjustment to childhood cancer. | No significant association found between income levels and higher risk of PTSD among PCS (p = ns). |
| Phipps | 2006 | USA | 162 PCS with various cancers, mean age at diagnosis 12.9 years, SD 3.0 were recruited from outpatient clinics of a major children’s cancer center. | No significant association found between socioeconomic status and any PTSS measures. |
| Stuber | 2010 | USA | 6542 PCS with various cancers, mean age at diagnosis 8.21 years, SD 5.87 were recruited as part of the Childhood Cancer Survivor Study. | Significant association between PTSD and annual income <$20,000 (95% CI= 1.21-2.20). |
| Tillery | 2019 | USA | 50 PCS with various cancers, mean age at diagnosis 2.15 years, SD 1.43 were recruited from outpatient clinics at a St Jude Children’s Research Hospital. | No significant association between PTSD and socioeconomic status. |
| Bemis | 2015 | USA | 151 PCS with various cancers, mean age at diagnosis 10.6 years, SD 3.9 were recruited from two paediatric hospitals in the US. | Significant association found between lower income levels and mother and child reported distress (p<0.05). |
| Phipps | 2009 | USA | 199 PCS with various cancers, mean age at data collection 12.38 years, SD 3 were recruited from outpatient clinics at a major paediatric oncology center in the USA. | No significant association found between SES or any of the PTSDI subscales or total score. |
| **Family Nucleus of Paediatric Cancer Survivors** | | | | |
| Brown | 2003 | USA | 52 mothers of PCS with various cancers, and 42 mothers of healthy children were recruited from a major-university affiliated medical centre in USA. Mean age of PCS at diagnosis was 9.5 years, SD 4.88. | No significant association was found between SES and PTSD symptoms. |
| Tillery | 2019 | USA | 50 parents of PCS with various cancers and 47 parents of healthy children were recruited from outpatient clinics at a St. Jude Children’s Research Hospital. Mean age of PCS at diagnosis was 4.57 years, SD 1.07. | No significant association found between income level and level of PTSS. |
| Yang | 2022 | China | 91 parents of PCS with various cancers, mean age 38.24 years, SD 6.30 and 96 parents of healthy children, mean age 38.80 years, SD 4.67, were recruited from the department of paediatrics of four general hospitals in southern China. Mean age of PCS at diagnosis was 10.92 years, SD 2.95. | Significant association found between lower monthly household income levels and parental PTSS (p<0.05). |

Abbreviations: Post-traumatic Stress Disorder, PTSD; Post-traumatic Stress Symptom, PTSS; Paediatric Cancer Survivors, PCS; Standard Deviation, SD; Confidence Interval, CI; Not significant, ns.
†Outcomes of interest include logistic or linear regression analysis for any association between social status and risk of PTSD and severity of PTSS

**Supplementary Table 8:** Quality assessment of included cohort studies using the Joanna Brigg’s Institute Critical Appraisal tool

| **Study** | **1** | **2** | **3** | **4** | **5** | **6** | **7** | **8** | **9** | **10** | **11** |
| --- | --- | --- | --- | --- | --- | --- | --- | --- | --- | --- | --- |
| Barakat | Y | Y | Y | Y | Y | Y | Y | Y | Y | NA | Y |
| Brown | Y | Y | Y | Y | Y | Y | Y | N | N | NA | Y |
| Gerhardt | N | Y | Y | Y | Y | U | Y | Y | Y | NA | Y |
| Phipps 2014 | N | Y | Y | Y | Y | U | Y | N | N | NA | Y |
| Schwartz | N | Y | Y | Y | Y | U | Y | Y | N | NA | Y |
| Yang | Y | Y | Y | Y | Y | Y | Y | N | N | NA | Y |
| Phipps 2006 | Y | Y | Y | Y | Y | Y | Y | N | N | NA | Y |
| Kazak | Y | Y | Y | Y | Y | U | Y | Y | Y | NA | Y |
| Tillery | Y | Y | Y | Y | Y | Y | Y | N | N | NA | Y |
| Stuber | Y | Y | Y | Y | Y | Y | Y | N | N | NA | Y |
| Seitz | Y | Y | Y | Y | Y | Y | Y | N | N | NA | Y |
| Bemis | Y | Y | Y | Y | Y | Y | Y | N | N | NA | Y |
| Bruce | Y | Y | Y | Y | Y | Y | Y | N | N | NA | Y |
| Clawson | N | Y | Y | Y | Y | U | Y | N | N | NA | Y |
| D’Urso | N | Y | Y | Y | Y | Y | Y | N | N | NA | Y |
| Ozono 2007 | Y | Y | Y | Y | Y | Y | Y | Y | Y | NA | Y |
| Phipps 2009 | N | Y | Y | Y | Y | Y | Y | Y | Y | NA | Y |
| Poder | Y | Y | Y | Y | Y | Y | Y | N | N | NA | Y |
| Baenziger | Y | Y | Y | Y | Y | Y | Y | Y | Y | NA | Y |
| McCarthy | Y | Y | Y | Y | Y | Y | Y | Y | Y | NA | Y |
| van Gorp | Y | Y | Y | Y | Y | Y | Y | Y | Y | NA | Y |

| Checklist |
| --- |
| 1. Were the two groups similar and recruited from the same population? |
| 2. Were the exposures measured similarly to assign people to both exposed and unexposed groups? |
| 3. Was the exposure measured in a valid and reliable way? |
| 4. Were confounding factors identified? |
| 5. Were strategies to deal with confounding factors stated? |
| 6. Were the groups/participants free of the outcome at the start of the study (or at the moment of exposure)? |
| 7. Were the outcomes measured in a valid and reliable way? |
| 8. Was the follow up time reported and sufficient to be long enough for outcomes to occur? |
| 9. Was follow up complete, and if not, were the reasons to loss to follow up described and explored? |
| 10. Were strategies to address incomplete follow up utilized? |
| 11. Was appropriate statistical analysis used? |

Legend:

Y – Yes

N – No

U – Unclear

NA – Not applicable

**Supplementary Figure 1:** Funnel plot for visual inspection of publication bias in studies assessing risk of PTSD in PCS


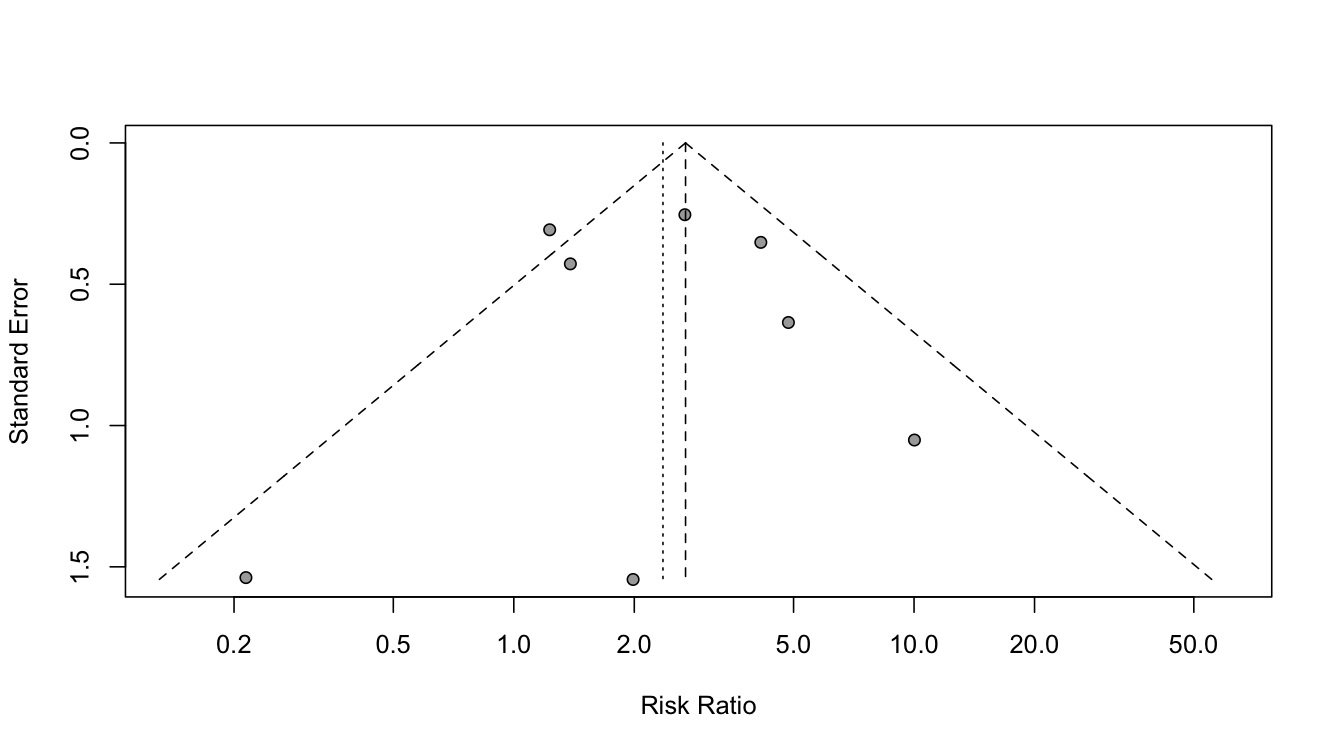


**Supplementary Figure 2:** Trim-and-fill analysis for publication bias in studies assessing risk of PTSD in PCS


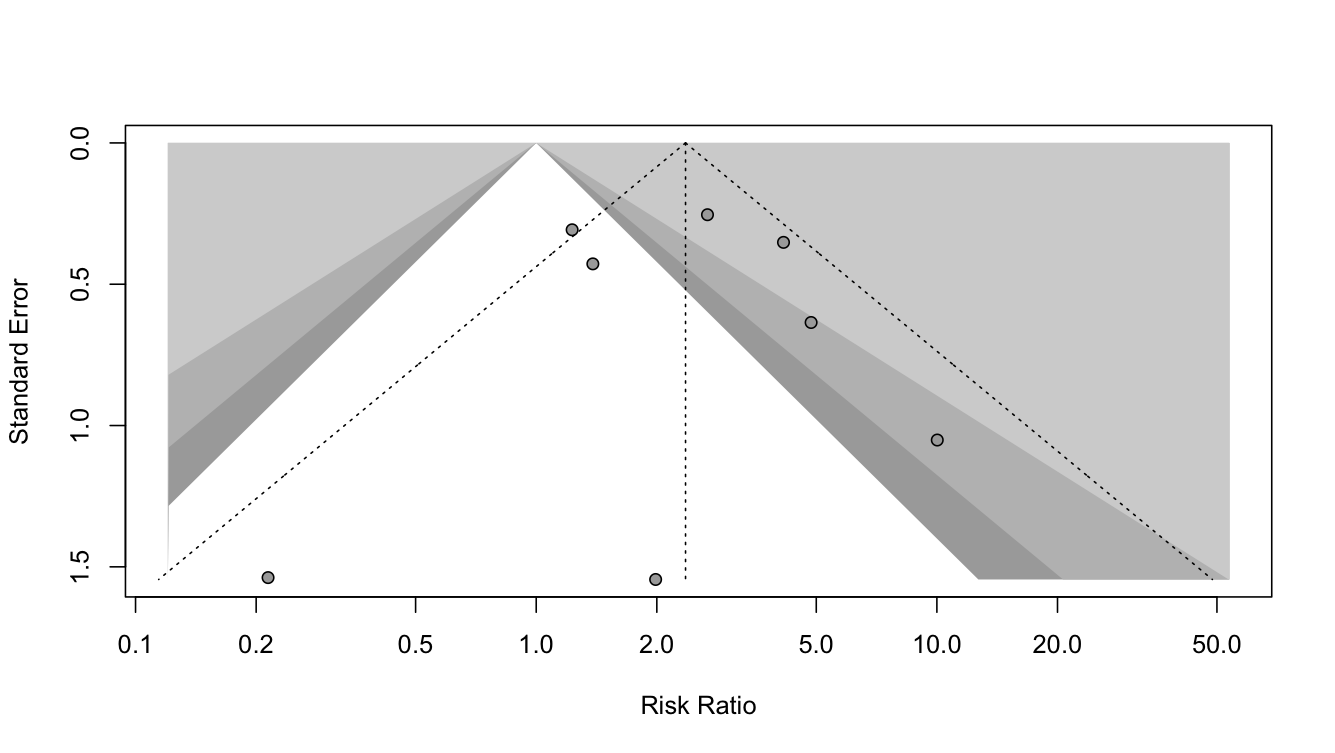


**Supplementary Figure 3:** Quantitative assessment publication bias in studies assessing risk of PTSD in PCS


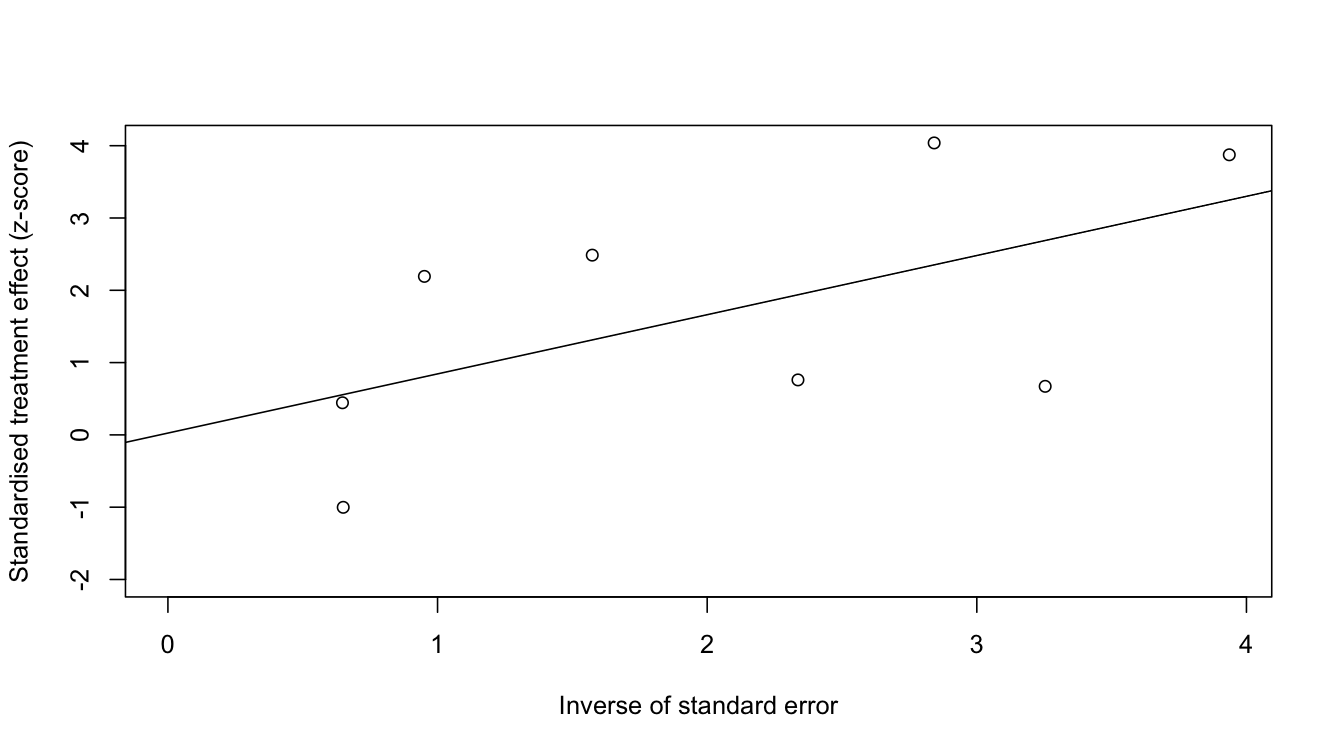


**Supplementary Figure 4:** Leave-one-out analysis of studies assessing the risk of PTSD in PCS, compared to non-cancer controls, using the random effects model


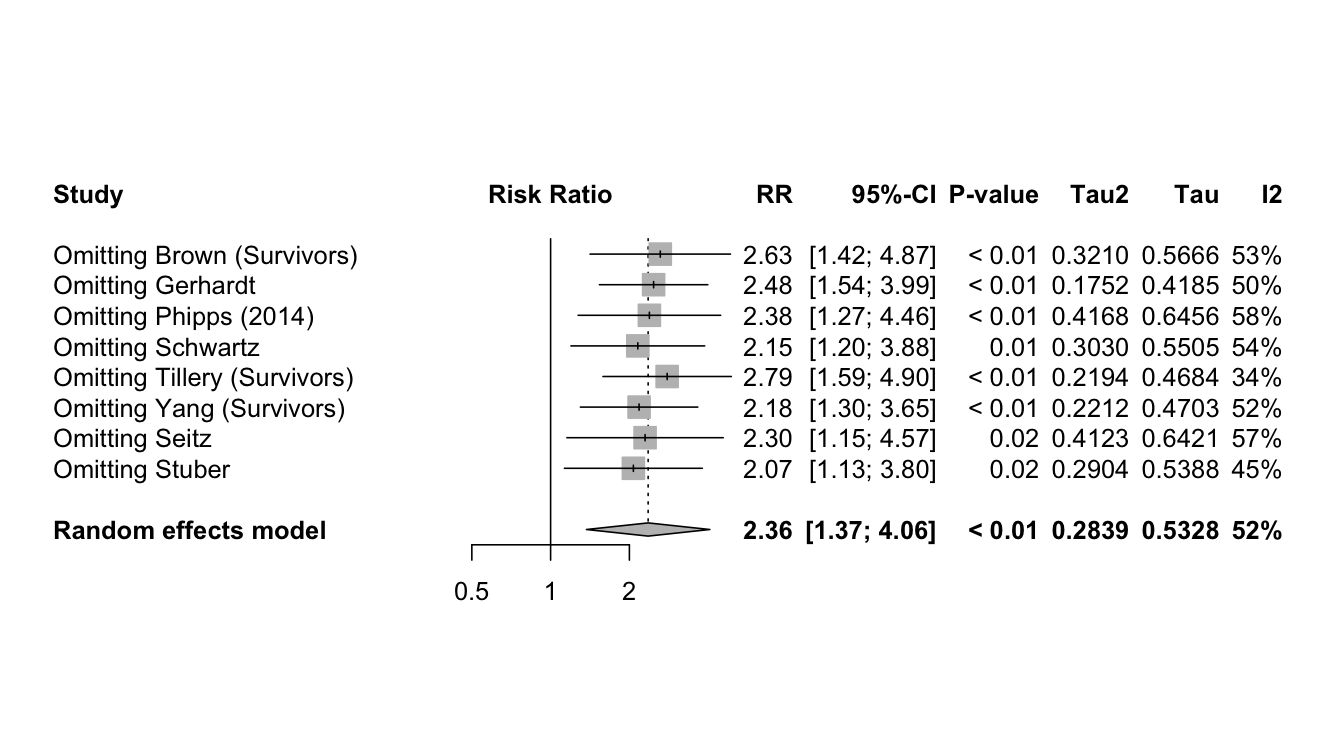


**Supplementary Figure 5:** Outlier assessment of studies assessing the risk of PTSD in PCS, compared to non-cancer controls, using the random effects model


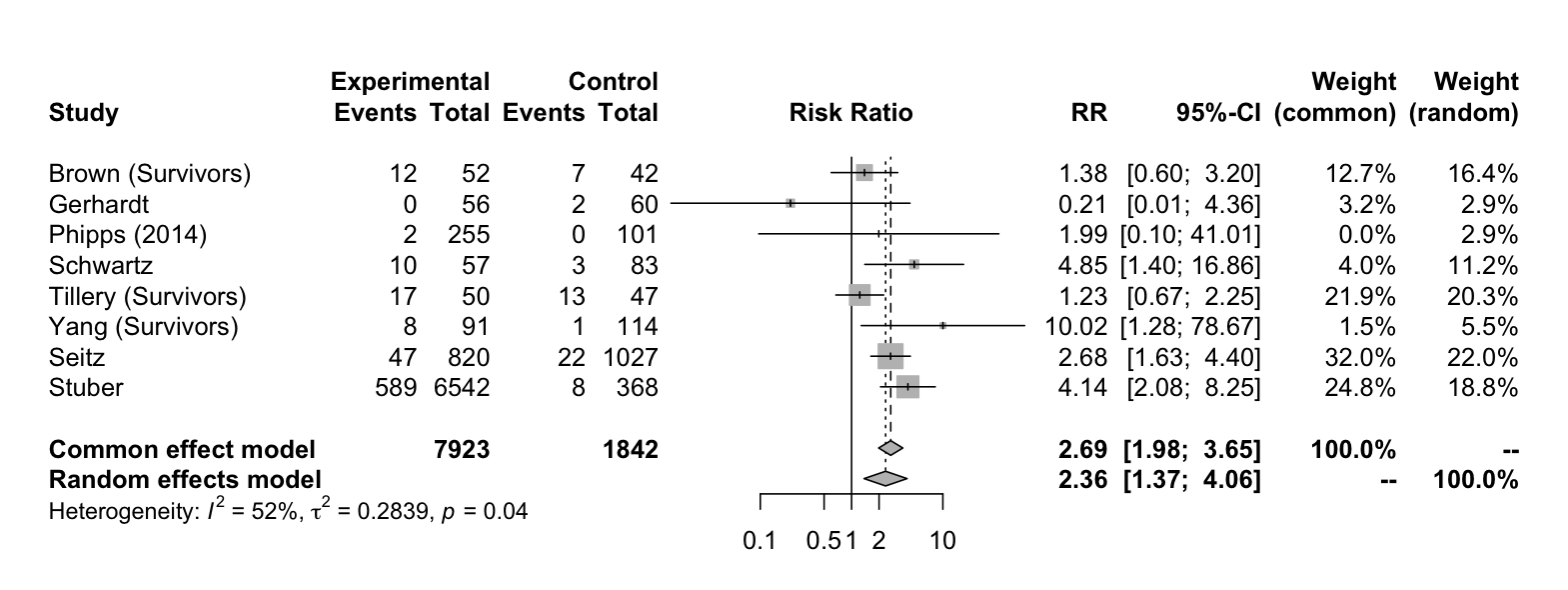


**Supplementary Figure 6:** Funnel plot for visual inspection of publication bias in studies assessing severity of PTSS in PCS


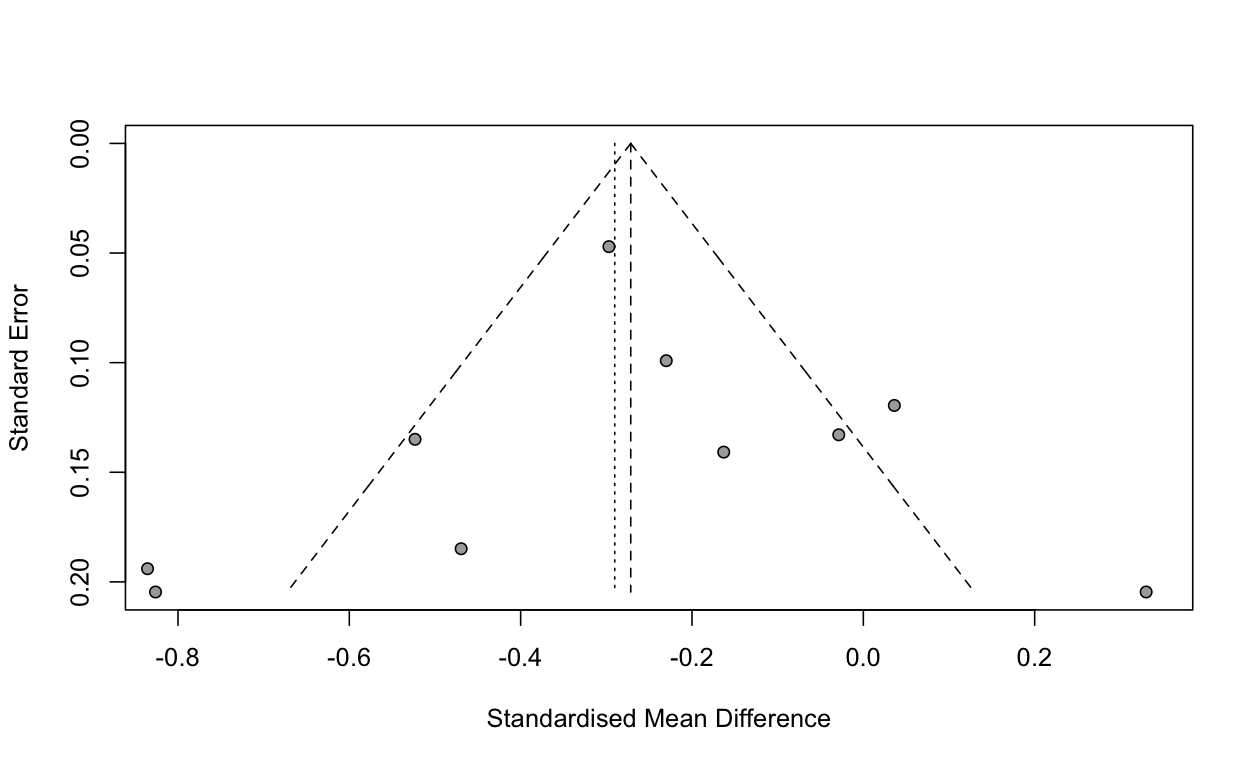


**Supplementary Figure 7:** Trim-and-fill analysis for publication bias in studies assessing severity of PTSS in PCS


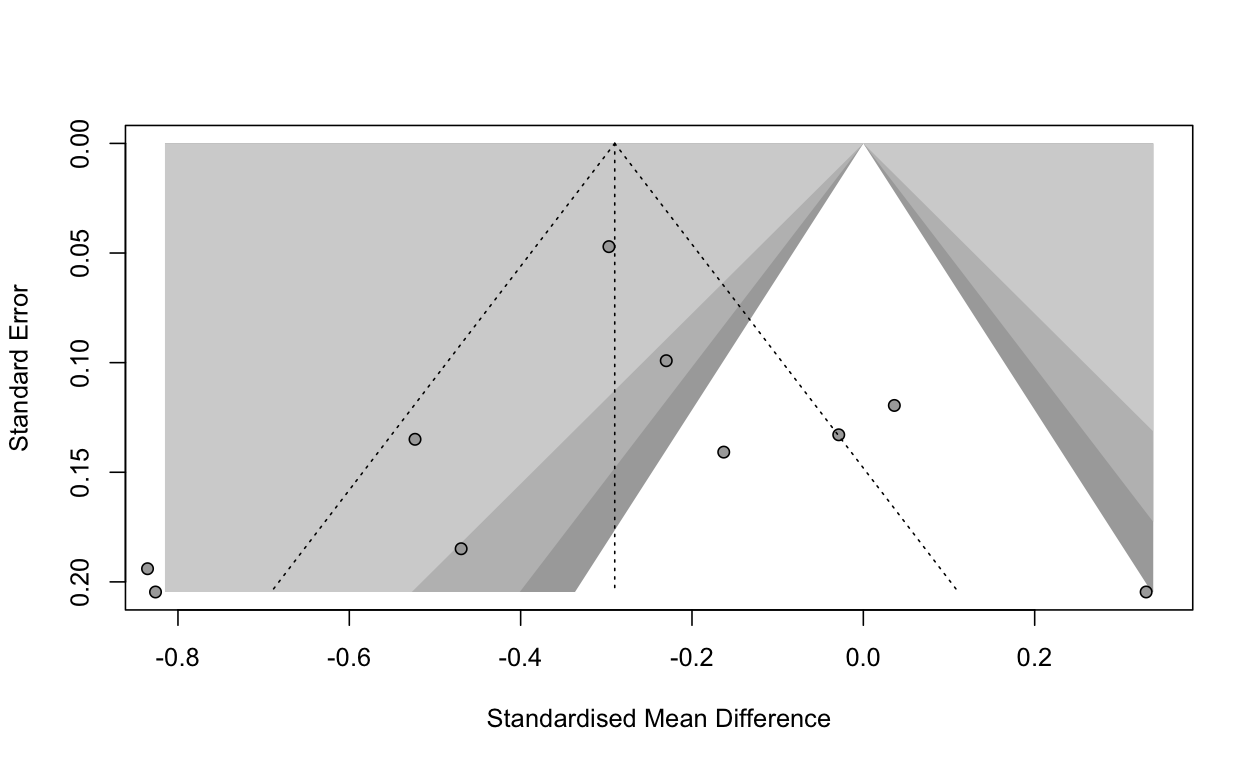


**Supplementary Figure 8:** Quantitative assessment publication bias in studies assessing severity of PTSS in PCS

**
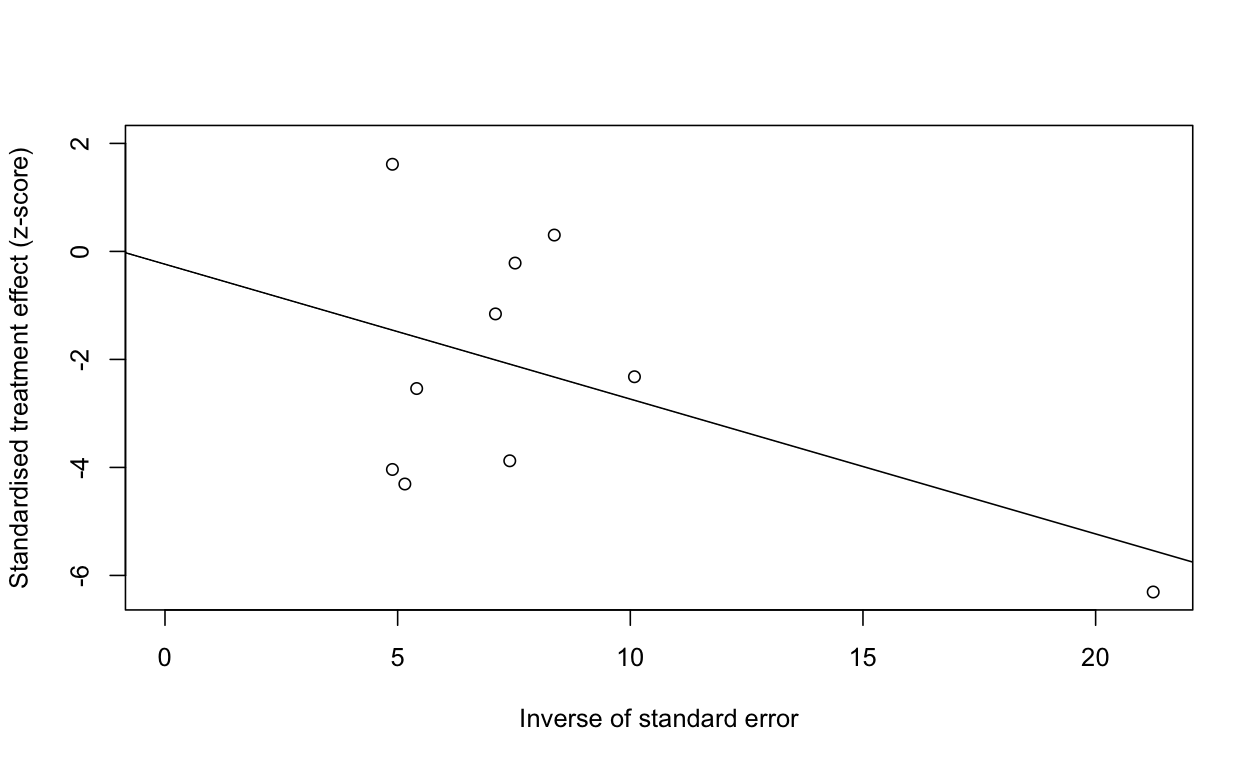
**

**Supplementary Figure 9:** Leave-one-out analysis of studies assessing the severity of PTSS in PCS, compared to non-cancer controls, using the random effects model


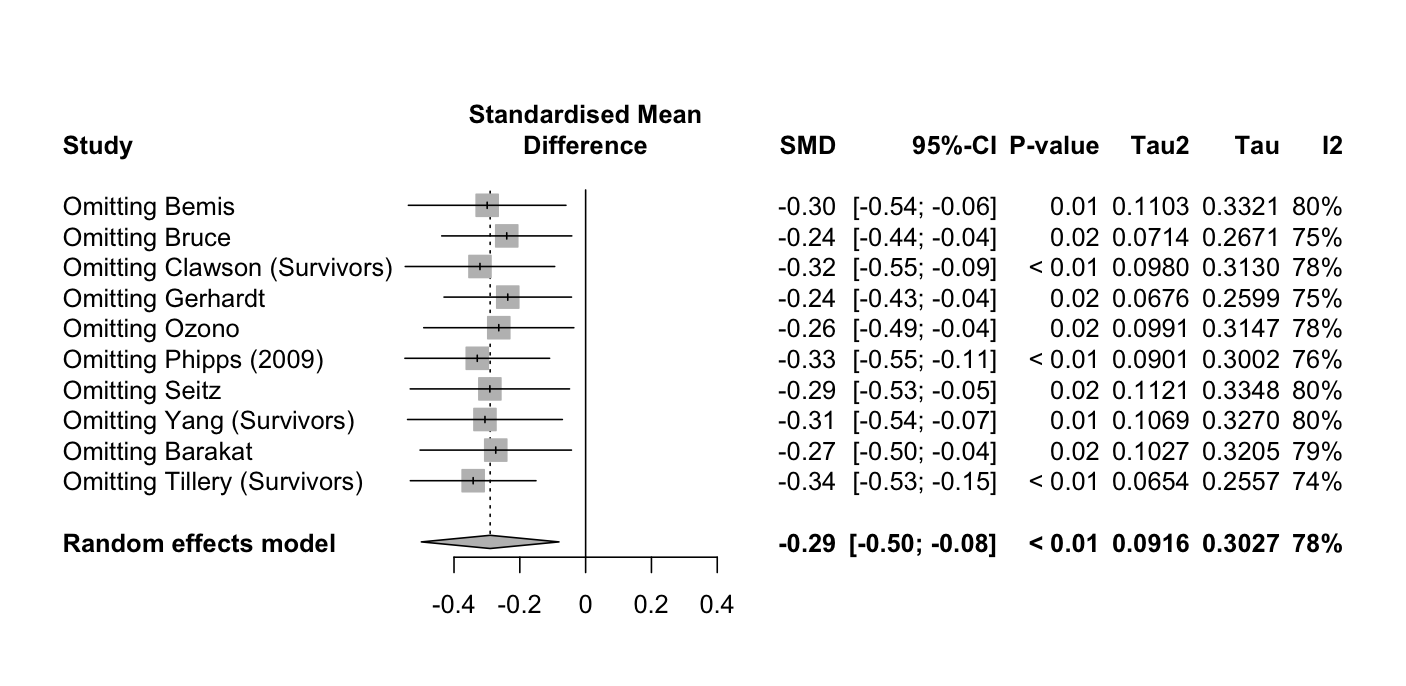


**Supplementary Figure 10:** Outlier assessment of studies assessing the risk of PTSD of PCS in PCS, compared to non-cancer controls, using the random effects model


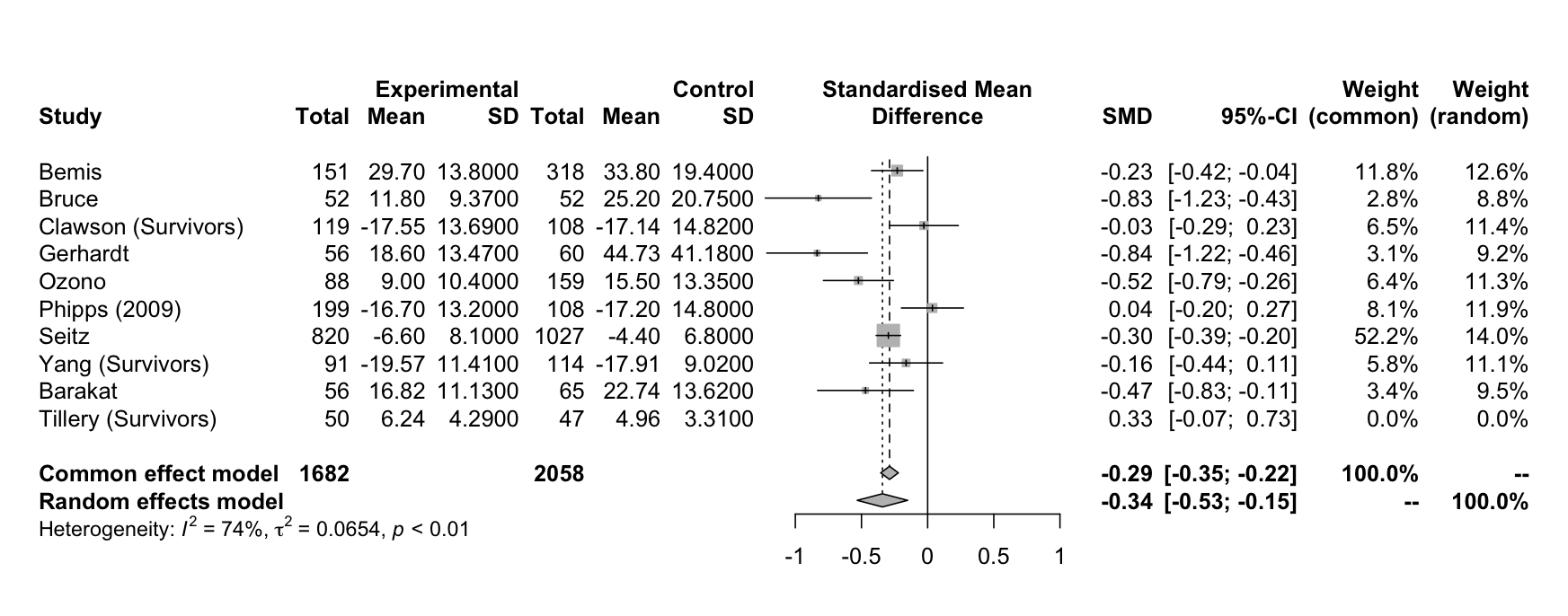

Supplement: Low et al. supplementary material [file S2056472424008056sup001.docx]
